# Overall Health Care Cost During the Year Following Diagnosis of Colorectal Cancer Stratified by History of Colorectal Evaluative Procedures

**DOI:** 10.1093/jcag/gwab001

**Published:** 2021-03-06

**Authors:** Lawrence Paszat, Rinku Sutradhar, Jin Luo, Linda Rabeneck, Jill Tinmouth, Nancy N Baxter

**Affiliations:** 1 Institute for Healthcare Policy, Management, and Evaluation, University of Toronto, Toronto, Ontario, Canada; 2 Cancer Research Program, ICES, Toronto, Ontario, Canada

**Keywords:** *Colonoscopy*, *Colorectal cancer*, *Fecal occult blood testing*, *Health care costs*

## Abstract

**Background:**

The cost-effectiveness of colorectal screening has been modeled; however, the cost of health care following the diagnosis of colorectal cancer has not been described stratified by history of colorectal evaluative procedures.

**Methods:**

We identified persons with first diagnosis of colorectal cancer between 2015 and 2017 from the Ontario Cancer Registry, and categorized them by history of colorectal evaluative procedures during Period 1 (the 10 years before the 6-month prediagnostic interval) with or without procedures during Period 2 (the 6 month prediagnostic interval), versus only during Period 2, versus none. We extracted overall health care cost 1 year following diagnosis from population-wide administrative databases.

**Results:**

Among cases diagnosed at 52 to 74 years, overall health care cost among those with no colorectal evaluative procedures on or before the date of diagnosis is $71,039.65 (SD $51,825.18), compared to $48,406.15 (SD $38,843.64) among those who received colorectal evaluative procedures during Period 1, with or without procedures during Period 2. Among the population aged 20 to 74 years at diagnosis, cases with ≥1 screening colonoscopies for hereditary CRC syndrome, the mean overall initial cost was between $32,300.32 (SD) and $33,084.67 (SD $39,905.77), and those with ≥1 screening colonoscopies because of a first-degree relative with CRC, was between $36,344.71 (SD $35,539.85) and $45,456.41 (SD $49,818.59).

**Conclusions:**

Overall health care cost is lower among cases who received colorectal evaluative procedures during Period 1, with or without procedures during Period 2, and among those with screening colonoscopy for hereditary CRC syndromes or affected first-degree relatives.

## Introduction

Opportunistic colorectal screening in Ontario began during the 1990s (1–3). Ontario inaugurated its population-based colorectal screening program ColonCancerCheck (CCC) in 2008, recommending to 50 to 74-year-old persons biennial screening with guaiac fecal occult blood testing (gFOBT) except for those with affected first-degree relatives, to whom screening colonoscopy was recommended (4). Frequent colonoscopy is recommended beginning at a young age for those with hereditary syndromes (5,6).

The cost-effectiveness of colorectal screening in reducing mortality from colorectal cancer (CRC) in Ontario has been demonstrated by modeling (7,8), but variation in health care cost following diagnosis of CRC has not been reported stratified by history of colorectal screening. The aim of this study is to estimate mean overall health care cost per case borne by the government of Ontario, during the year following diagnosis of CRC, in the screening age-eligible population aged 52 to 74 years at diagnosis between 2015 and 2017, stratified by history of colorectal screening or evaluative procedures, and in the population diagnosed at age 20 to 74 years, by history of screening colonoscopy. The usefulness of this information would be primarily as inputs in modeling cost-effectiveness of interventions, including but not limited to, colorectal screening, and other policy questions focused on CRC control.

## METHODS

### Identification of CRC Cohort in the Screening-Eligible Age Range 52 to 74 Years

To estimate the mean overall cost of care for CRC, we identified persons aged between 52 and 74 years at first diagnosis of CRC between 2015 and 2017 from the Ontario Cancer Registry (OCR). We excluded cases with previous diagnosis of CRC, any other invasive malignancy within 5 years before, or 1 year following, diagnosis of CRC, but did not exclude cases with multiple synchronous CRCs on the same diagnosis date or metachronous CRC diagnosed within 12 months from the index diagnosis date. We excluded cases with missing cancer stage. We identified deaths from the Registered Persons Database. We categorized cases broadly as colon versus rectosigmoid+ rectal, because of potential misclassification in registry and administrative data. Date of last follow-up was March 31, 2019.

We extracted billing claims for colonoscopy from the Ontario Health Insurance Plan (OHIP) database during 10.5 years before diagnosis, and records gFOBT 10.5 years before diagnosis, from the OHIP database, and after April 1, 2008, from the ColonCancerCheck (CCC) database of gFOBT reports. The indication for colonoscopy is not recorded. The indication for gFOBT is unknown in the OHIP database; gFOBT records in the CCC database should represent screening of asymptomatic individuals, however, CCC does not verify the asymptomatic status of those completing gFOBT.

The records of gFOBT and/or colonoscopy during Period 1 (the 10 years before the 6 month prediagnostic interval) would represent either diagnostic investigation, periodic colorectal screening or periodic postpolypectomy surveillance. We also identified gFOBT and/or colonoscopy during (Period 2) (the 6 month prediagnostic interval). The importance of the distinction between Period 1 and Period 2 derives from several issues including firstly, the inability to distinguish screening from diagnostic investigation in either period, secondly, the negative predictive value of colorectal evaluative procedures not leading to diagnosis of CRC during Period 1, which have effectively screened the patient, regardless of the intention or indication for the procedure, and thirdly, the prevalent nature of cancers detected without a prior history of colorectal evaluation during Period 1 and the detection bias associated with cases of cancer detected by the case’s first ever screening test during Period 2. It is possible, but not verifiable, that few persons with colonoscopy in both Periods had a postcolonoscopy CRC, as well as a few persons with colonoscopy in Period 1 but no colonoscopy in Period 2.

To categorize comorbidity, we used the Ambulatory Care Group program (9) to identify each of the eight major adult Ambulatory Diagnostic Group (ADG) categories, extracting records from the Canadian Institute for Health Information (CIHI) databases (Discharge Abstract Database [DAD], Same Day Surgery Database [SDS] and the National Ambulatory Care Record System Database [NACRS]) plus the OHIP database. Each case was categorized has having one or more of the eight major adult ADGs versus none.

### Identification Cohort of CRC Aged 20 to 74 Years to Examine Screening Colonoscopy Among Those With Hereditary CRC Syndromes or Affected First-Degree Relatives

We identified first diagnoses of CRC in the 20 to 74 age range from the OCR, excluding those with CRC at any time before 2015, those with missing stage, those with diagnosis of inflammatory bowel disease or with total colectomy before diagnosis of CRC, those whose residence was not in Ontario at the time of diagnosis and those with less than 36 months continuous eligibility for OHIP.

We identified billing claims for colonoscopy Period 1 (10 years before the 6 months before diagnosis), and during Period 2. We categorized them according to feecode in a hierarchical fashion in the following sequence: firstly, Z494 screening colonoscopy for persons with hereditary CRC syndrome (although this code may also be applied to persons with inflammatory bowel disease, we have already excluded such persons), secondly, Z499 screening colonoscopy for persons with affected first-degree relatives but not hereditary CRC syndromes, and thirdly, all other colonoscopies. The mean number of colonoscopies during Period 1 was computed, with the percent of cases with Stage 1 CRC.

### Identification of Health Care Costs Borne by the Government of Ontario

Costs for all health care services used by each case, regardless of indication, were extracted from linked databases held at ICES, beginning on the date of diagnosis, using algorithms originally developed by Wodchis et al. (10) and subsequently enhanced (11–16). Costs include inpatient, outpatient and emergency department hospital costs, visits and treatments at ambulatory cancer and dialysis centres, intravenous chemotherapy, radiation therapy, reimbursement of physicians and surgeons, medical laboratory services, oral medications including oral chemotherapy, in-patient rehabilitation, institutional complex continuing and long-term care, home care services and assistive device procurement. The data sources do not allow accurate separation of costs related to the initial care for CRC from costs incurred because of concurrent conditions. Costs were presented in 2017 Canadian dollars; on average during 2017, $ 1 United States currency = $1.2986 Canadian currency (17).

### Approximating the Mean Overall Health Care Cost During the Year Following Diagnosis of CRC Among Cases Aged 52 to 74 Years

We computed the mean overall health care cost across 360 days beginning on the date of diagnosis of CRC, to approximate the cost incurred during the first year following diagnosis. We re-computed the mean overall health care cost across the first 360 days, excluding cases with ≤690 days of follow-up (to approximate the mean overall cost during the entire year following diagnosis of CRC without contribution from cost incurred during the final year of life).

We stratified the mean overall health care cost across 360 days, beginning on the date of diagnosis, by the history of colorectal evaluative procedures firstly, during Period 1, plus or minus during Period 2, secondly, first ever procedures only during Period 2, and thirdly, no procedures on or before the date of diagnosis The mean overall health care cost was also stratified by age group at diagnosis (52 to 64 years versus 65 to 74 years), sex, anatomic site (colon versus rectosigmoid+rectum) and cancer stage.

Approximating the mean overall health care cost during the year following diagnosis of CRC among cases first diagnosed at age 20 to 74 years.

The method of computation was identical to that used with the 52 to 74-year-old cohort, however, stratification was by colonoscopy, using the hierarchy of firstly, screening colonoscopy for persons with hereditary CRC syndromes during Period 1, plus or minus during Period 2, or only during Period 2, secondly, screening colonoscopy for persons with affected first-degree relatives but not hereditary CRC syndromes during Period 1, plus or minus during Period 2, or only during Period 2, thirdly, other colonoscopy during Period 1, plus or minus during Period 2, or only during Period 2, or fourthly, no colonoscopy on or before the date of diagnosis of CRC.

## RESULTS

### Cohort of Persons With CRC Aged 52 to 74 Years

We identified 12,550 cases of CRC aged 52 to 74 years between 2015 and 2017 from the OCR. We excluded 570 cases because of previous diagnosis of CRC, 752 cases because of any other invasive malignancy within 5 years before diagnosis, 344 cases with any other invasive malignancy 1 year following the diagnosis of CRC, 903 cases lacking cancer stage, and 4 cases because the recorded diagnosis date fell after the recorded date of death, leaving a cohort of 9977 cases (comprising 6310 persons with colorectal evaluative procedures during Period 1 with or without procedures during Period 2, 2545 persons with procedures only during Period 2, and 1122 persons with no procedures at any time on or before the date of diagnosis.

The cohort is described by history and timing of first gFOBT and/or colonoscopy in Table 1, and by history and timing of first gFOBT and/or colonoscopy stratified by age, and sex, for each stage of colon cancer and each stage of rectosigmoid+rectal cancer in Table 2. Among cases with colorectal evaluative procedures during Period 1, 3424/6310 (54.3%) underwent colonoscopy but no gFOBT during Period 2 and 1543/6310 (24.5%) underwent gFOBT plus colonoscopy, while 1343/6310 (21.3%) did not have colonoscopy during the 6-month prediagnostic interval. Cases with colorectal evaluative procedures during Period 1 were older and were more likely to have one or more major adult ADG diagnoses compared to those who did not. The percent of cases diagnosed with stage 1 CRC is highest among those who began to receive colorectal evaluative procedures during Period 1, conversely the per cent diagnosed with stage 4 CRC is highest among those with no record of gFOBT or colonoscopy on or before the date of diagnosis (Table 2).

**Table 1.** History of colorectal evaluative procedures among newly diagnosed cases of CRC 2015–2017 aged 52–74 years

Colorectal evaluative procedures, during Period 1 (10 years before the 6 month prediagnostic interval) *n* = 6310		First ever colorectal evaluative procedure during Period 2 *n* = 2545	No procedures *n* = 1122
Colorectal evaluative procedures during Period 1	Colorectal evaluative procedures during Period 2	Colorectal evaluative procedures during Period 2	Not applicable
≥ 1 colonoscopy no FOBT *n* = 1089/6310 (17.2%)	Colonoscopy no FOBT 752/1089 (69.1%) FOBT + colonoscopy 82/1089 (7.5%) No FOBT or colonoscopy 255/1089 (23.4%)	Colonoscopy no FOBT *n* = 1862/2545 (73.2%) FOBT + colonoscopy*n* = 598/1862 (23.5%) FOBT no colonoscopy *n* = 86/2545 (3.3%)	Not applicable
≥ 1 colonoscopy + ≥1 FOBT*n* = 1562/6310 (24.8%)	Colonoscopy no FOBT 983/1562 (62.9%) FOBT + colonoscopy 234/1562 (15.0%) FOBT no colonoscopy 35/1562 (2.2%) No FOBT or colonoscopy 310/1562 (19.9%)		
≥1 FOBT (no colonoscopy) *n* = 3659/6310 (58.0%)	Colonoscopy no FOBT 1689/3659 (46.2%) FOBT + colonoscopy 1227/3659 (33.5%) FOBT no colonoscopy 122/3659 (3.3%) No FOBT or colonoscopy 621/3659 (17.0%)		

**Table 2.** Age, sex, cancer stage and anatomic site according to history of colorectal evaluative procedures during Period 1 (10 years before the 6-month prediagnostic interval) and Period 2 (6 months before diagnosis)

	Colorectal evaluative procedures during Period 1 (includes periodic screening, surveillance or diagnosis of symptoms) *n* = 6310		First ever colorectal evaluative procedure during Period 2 *n* = 2545			No gFOBT or colonoscopy on or before diagnosis date *n* = 1122
	*n* = 1089 Colonoscopy but no gFOBT during Period 1	*n* = 5221 gFOBT ± colonoscopy during Period 1				
	Colonoscopy no FOBT *n* = 752/1089 (69.1%) FOBT + colonoscopy *n* = 82/1089 (7.5%) no FOBT or colonoscopy *n* = 255/1089 (23.4%)	Colonoscopy no FOBT 2672/5221 (51.2%) FOBT + colonoscopy 1461/5221 (28.0%) FOBT no colonoscopy 157/5221 (2.2%) no FOBT or colonoscopy 931/5221 (17.8%)	Colonoscopy no FOBT 1862/2545 (73.2%)	FOBT + colonoscopy 598/2545 (23.5%)	FOBT no colonoscopy 86/2545 (3.3%)	
Age Mean (SD) Median (IQR)	64.01 years (SD 6.34 years) 65 years (IQR 59–69 years)	65.40 (SD 6.05 years) 66 years (IQR 61–70 years)	62.35 years (SD 6.52 years) 62 years (IQR 57–62 years)		62.83 years (SD 6.61 years) 63 years (IQR 57–68 years)	63.11 years (SD 6.54 years) 63 years (IQR 58–69 years)
Sex Female Male	480 (44.1%) 609 (55.9%)	2,270 (43.5%) 2,951 (56.5%)	636 (34.2%) 1225 (65.8%)		215 (31.4%) 469 (68.6%)	439 (39.1%) 683 (60.9%)
≥1 Major Adult ADG Yes No	782 (71.8%) 307 (28.2%)	3332 (63.8%) 1889 (36.2%)	1072 (57.6%) 789 (42.4%)		359 (52.5%) 325 (47.5%)	635 (56.6%) 487 (43.4%)
Stage 1	324 (31.4%)	1543 (29.6%)	450 (24.2%)		178 (26.0%)	64 (5.7%)
Stage 2	250 (23.0%)	1216 (23.3%)	413 (22.2%)		161 (23.5%)	274 (24.4%)
Stage 3	325 (29.8%)	1614 (30.9%)	674 (36.2%)		244 (35.7%)	315 (28.1%)
Stage 4	172 (15.8%)	848 (16.2%)	324 (17.4%)		101 (14.8%)	469 (41.8%)
Anatomic site Colon Rectosigmoid + Rectum	800 (73.5%) 289 (26.5%)	3542 (67.8%) 1679 (32.2%)	985 (52.9%) 876 (47.1%)		413 (60.4%) 271 (39.6%)	781 (69.6%) 341 (30.4%)

### Cohort of Persons With CRC Aged 20 to 74 Years to Examine Screening Colonoscopy for Hereditary CRC Syndromes or Affected First-Degree Relatives

This cohort includes 1598/11,547 (13.8%) persons aged 20 to 49 years, among whom 62/1598 (3.9%) had received screening colonoscopy because of a hereditary CRC syndrome or affected first-degree relatives and 120/1598 (7.5%) had received colonoscopy for other indications during Period 1. Among persons aged 50 to 64 years at diagnosis, 24/5061 (0.5%) had received screening colonoscopy because of a hereditary CRC syndrome, 194/5061 (3.8%) because of affected first-degree relatives and 1009/5061 (20.0%) had received colonoscopy for other indications during Period 1, compared to those aged 65 to 74 years, among whom 23/4888 (0.5%) had received screening colonoscopy because of a hereditary CRC syndrome, 171/4888 (3.5%) because of affected first-degree relatives and 1421/4888 (29.1%) for other indications during Period 1. The per cent of persons with colonoscopy during Period 2, and the number (percent) of those diagnosed with stage 1 CRC by colonoscopy history are presented in Table 3.

**Table 3.** History of colonoscopy among newly diagnosed cases of colorectal cancer 2015–2017 at ages 20–74 years

	*n*	Colonoscopies during Period 1 (10 years before > the 6-month prediagnostic interval)	Colorectal evaluative procedures during Period 2 (the 6-month prediagnostic interval)	Stage 1 at diagnosis
Overall	11,547	Mean (SD) 0.32 (SD 0.76) Median (IQR) 0 (IQR 0-0)	8590/11,547 colonoscopy	3048/11,547 (26.4%)
First screening colonoscopy for hereditary syndrome during Period 1 (likely periodic screening or surveillance)	18	Mean (SD) 4.39 (SD 2.33) Median (IQR) 5 (IQR 2–6)	17/18 colonoscopy	11/18 (61.1%)
First screening colonoscopy for hereditary syndrome during the Period 2	38	Mean (SD) 0.97 (SD 1.24) Median (IQR) 1 (IQR 0–2)	38/38 colonoscopy	20/38 (52.6%)
First screening colonoscopy for affected first-degree relative during Period 1 (likely periodic screening or surveillance)	112	Mean (SD) 1.75 (SD 0.94) Median (IQR) 2 (IQR 1–2)	76/112 colonoscopy 36/112 no colonoscopy	33/76 (43.3%) 12/36 (33.3%)
First screening colonoscopy for affected first-degree relative during Period 2	306	Mean (SD) 0.36 (SD 0.69) Median (IQR) 0 (IQR 0–1)	306/306 colonoscopy	161/306 (52.6%)
First other colonoscopy >6 months during Period 1 (likely periodic screening, surveillance, or diagnostic evaluation of symptoms)	2,550	Mean (SD) 1.28 (SD 1.01) Median (IQR) 1 (IQR 1–2)	2103/2550 colonoscopy	805/2550 (31.6%)
First other colonoscopy ≤6 months during Period 2	6,045	Mean 0 Median 0	6045/6045 colonoscopy	1698/6045 (28.1%)
No colonoscopy on or before diagnosis	2,478	Mean 0 Median 0	0	308/2478 (12.4%)

### Mean Overall Health Care Cost During the First Year Following Diagnosis of CRC Among Persons Aged 52 to 74 Years

Mean overall health care cost across the first 360 days in 2017 Canadian dollars is $51,883.14 (standard deviation [SD] $41,151.89), which is an approximation of the cost during the first year following diagnosis. The number whose follow-up time extended >690 days after diagnosis is 7882/9997 (79.0%), whose mean overall cost is $47,823.43 (SD $37,445.49); by definition, this mean overall health care cost does not include any cost incurred during the 360 days before death (Table 4).

**Table 4.** Mean overall initial cost of CRC 2015–2017 age 52–74 years

	*N*	Mean overall initial cost (SD) including all cases	Mean overall initial cost (SD) among those alive > 330 days	Mean overall initial cost (SD) among those alive > 690 days
Mean overall cost (SD)	9977	$51,883.14 (SD $41,151.89)	$51,065.61 (SD $40,298.33)	$47,823.43 ($37,445.49)
Stratified mean overall cost (SD)				
Colorectal evaluative procedures during Period 1 +/- Period 2	6310	$48,406.15 (SD $38,843.64)	$47,333.96 (SD $38,278.90)	$44,330.26 ($34,788.07)
First ever colorectal evaluative procedure during Period 2	2545	$52,058.46 (SD $39,031.02)	$51,895.44 ($38,552.33)	$49,536.00 ($37,485.32)
No FOBT and/or colonoscopy on or before the date of diagnosis	1122	$71,039.65 (SD $51,825.18)	$75,569.37 ($49,747.87)	$71,150.25 ($48,334.33)
Stage 1 Colon	1611	$24,832.55 (SD $28,916.53)	$24,344.55 (SD $27,717.30)	$23,446.68 (SD $25,690.61)
Stage 1 Rectosigmoid + rectum	966	$29,432.81 (SD $32,611.74)	$28,816.56 (SD $31,587.13)	$27,976.25 (SD $28,373.18)
Stage 2 Colon	1709	$39,186.22 (SD $41,809.42)	$37,879.74 (SD $39,635.54)	$36,758.49 (SD $37,554.91)
Stage 2 Rectosigmoid + rectum	605	$58,194.65 (SD $42,840.22)	$56,639.51 (SD $38,324.07)	$54,734.53 (SD $33,270.74)
Stage 3 Colon	1878	$60,704.89 (SD $33,117.19)	$60,478.88 (SD ($32,991.44)	$58,334.10 (SD $29,443.51)
Stage 3 Rectosigmoid + rectum	1294	$71,339.89 (SD $34,875.72)	$71,223.83 (SD $34,198.35)	$70,684.63 (SD $32,800.60)
Stage 4 Colon	1323	$72,032.39 (SD $41,894.82)	$84,354.32 (SD $36,783.66)	$81,636.69 (SD $36,829.80)
Stage 4 Rectosigmoid + rectum	591	$76,831.19 (SD $40,528.60)	$90,105.08 (SD $35,310.81)	$87,189.51 (SD $30,865.23)
Age 52 - 64	4799	$53,326.80 (SD $41,577.06)	$52,608.16 (SD $40,468.33)	$49,526.14 (SD $38,028.84)
Age 65 - 74	5178	$50,545.14 (SD $40,711.22)	$49,583.68 (SD $40,082.95)	$46,193.45 (SD $36,809.41)
Females	4040	$49,825.58 (SD $39,292.23)	$48,355.63 (SD $37,795.23)	$45,236.01 (SD $34,596.25)
Males	5937	$53,283.26 (SD $42,315.98)	$52,926.26 (SD $41,832.53)	$49,633.18 (SD $39,218.17)

Stratifying mean overall health care cost by history of colorectal evaluative procedures, among those with a procedures during Period 1, plus or minus during Period 2 (6310/9977; 63.2%) the cost is lowest, $48,406.15 (SD $38,843.64), compared to $52,058.46 (SD $39,031.02) among those whose first ever procedure occurred during Period 2 (2545/9977; 25.5%), and $71,039 (SD 51,825.18) among those with neither gFOBT nor colonoscopy at any time on or before diagnosis. Stratifying mean overall health care cost by both stage and anatomic site (colon versus rectosigmoid + rectal) produces a wide range of mean overall health care cost, from $24,832.55 (SD $28,916.53) for Stage 1 colon cancer (ICD10 C18) to $76,831.19 (SD $40,528.60) for Stage 4 rectosigmoid + rectal cancer. Mean overall health care cost for Stage 4 cases are higher for those living >330 days: mean overall health care cost for Stage 4 rectosigmoid + rectal cancers rises to $90,105.08 (SD $35,310.81).

### Overall Health Care Cost During the First Year Following Diagnosis of CRC Among Cases Aged 20 to 74 Years Stratified by Screening Colonoscopy

Among cases with ≥1 screening colonoscopies for hereditary CRC syndrome, the mean overall health care cost was between $32,300.32 (SD) and $33,084.67 (SD $39,905.77), and those with ≥1 screening colonoscopy because of a first-degree relative with CRC, was between $36,344.71 (SD $35,539.85) and $45,456.41 (SD $49,818.59). Cases with colonoscopy for other indications had fewer prior colonoscopies and higher mean overall health care cost, between $48,490.07 (SD $39,544.86) and $48,904.68 (SD $37,023.46). The mean overall health care cost for cases without colonoscopy at any time on or before diagnosis was $65,603.92 ($48,351.97) (Table 5). We are unable to present mean overall health care costs simultaneously stratified by both colonoscopy history and age at diagnosis because of small cell counts; however, the cost is highest for those aged 20 to 49 years ($54,470.68 [SD $41,771.24] and lowest among aged 65–74 ($50,501.20 [SD] $50,496.44). The number of cases aged 65 to 74 years in this colonoscopy-stratified cohort is lower than in the cohort aged 52 to 74 years because of the additional exclusion criteria, however, the estimates of mean overall health care cost are similar for the two cohorts (Tables 4 and 5).

**Table 5.** Mean overall initial cost of CRC 2015–2017 cases age 20–74 years stratified by likely screening colonoscopy during Period 1 compared to first ever colonoscopy in Period 2 or no colonoscopy

	*N*	Mean overall cost (SD)	Mean overall initial cost (SD) among those alive >330 days	Mean overall initial cost (SD) among those alive > 690 days
Overall	11,547	$51,951.72 (SD $41,016.33)	$51,056.56 (SD $40,100.33)	$47,768.23 (SD $37,157.38)
First colonoscopy for hereditary syndrome during Period 1 (likely periodic screening or surveillance)	18	$33,084.67 (SD $39,905.77)	$32,909.59 (SD $41,126.80)	$23,247.60 (SD $14,419.44)
First colonoscopy for hereditary syndrome during Period 2	38	$32,300.32 (SD $26,400.55)	$32,710.38 (SD $26,641.75)	$32,710.38 (SD $26,641.75)
First colonoscopy for affected first-degree relative during Period 1 (likely periodic screening or surveillance) *Subset with colonoscopy during Period 2* *Subset without colonoscopy during Period 2*	112 *76* *36*	$45,456.41 (SD $49,818.59) *$40,244.16 (SD $37,456.59)* *$56,460.09 (SD $68,419.23)*	$39,265.12 (SD $36,186.23) *$37,997.04 (SD $35,977.60)* *$42,215.23 (SD $37,085.05)*	$37,603.52 (SD $33,286.79) *$36,215.75 (SD $31,540.88)* *$40,857.59 (SD $37,453.57)*
First colonoscopy for affected first-degree relative during Period 2	306	$36,344.71 (SD $35,539.85)	$35,250.91 (SD $35,030.55)	$33,279.59 (SD $31,125.72)
First other colonoscopy during Period 1 (likely periodic screening, surveillance, or diagnosis of symptoms)	2550	$48,490.07 (SD $39,544.86)	$47,249.81 (SD $39,105.17)	$43,407.46 (SD $34,683.42)
First other colonoscopy during Period 2	6045	$48,905.68 (SD $37,023.46)	$48,413.63 (SD $ 36,466.96)	$46,497.48 (SD $34,741.06)
No colonoscopy on or before diagnosis	2478	$65,603.92 (SD $48,351.97)	$67,428.76 (SD $47,751.54)	$62,091.90 (SD $45,340.73)

## Discussion

Overall health care cost during the year following diagnosis of CRC diagnosed in the screening age-eligible population aged 52 to 74 years, is lower among cases who received colorectal evaluative procedures during Period 1 (the 10 years before the 6-month prediagnostic interval), generally with further evaluation during Period 2 (the 6-month prediagnostic window), reflecting the likelihood that some of those cases had participated in periodic screening. Although we cannot distinguish asymptomatic screening from diagnostic investigation as the intention of any one record of gFOBT or colonoscopy, it is likely that many had been participating in periodic asymptomatic screening by gFOBT or colonoscopy, or periodic colonoscopic surveillance after prior polypectomy, in addition to those who were undergoing diagnostic investigation of symptoms on one or more occasions. In effect, these cases have been screened, regardless of the intention and indication for those procedures. The steady increments in mean overall health care cost during the year following diagnosis of CRC among strata simultaneously defined by cancer stage and anatomic site of primary CRC indicate that initial care year costs should be reported by this simultaneous stratification and not by simple stratification by stage or by anatomic site of primary CRC.

The higher overall health care cost among those without colorectal evaluative procedures during Period 1 is not due to higher burden of comorbidity: The percent of cases with one or more major adult ADGs is highest among those with colorectal evaluative procedures during Period 1, who have the lowest cost during the year following the diagnosis of CRC. This supports the hypothesis that periodic screening, surveillance, or diagnostic investigation during Period 1 results in a higher percent of cases diagnosed in stage 1 and lower overall health care costs during the year following diagnosis.

Overall health care cost during the year following diagnosis of CRC between the ages of 20 to 74 years is lower among those who have had screening colonoscopies because of hereditary CRC syndromes or affected first-degree relatives. However, the higher CRC stage and higher overall mean cost for those with screening colonoscopy because of affected first-degree relatives in Period 1 who did not have colonoscopy in Period 2 illustrates that some postcolonoscopy CRCs will be associated with higher costs. The feecode for screening colonoscopy because of affected first-degree relatives was introduced at the end of 2011, so the maximum interval between the first family history colonoscopy and the diagnosis of CRC would have been 3 to 6 years, depending on the year of diagnosis.

There have been publications of health care costs in Ontario during the year following diagnosis of CRC among cohorts diagnosed before the 2015 to 2017 observation period. For cases diagnosed in 2009, de Oliveira et al. (11) estimated the mean cost per case to be $37,015 in 2009 Canadian dollars, and subsequently stratified costs for the 2009 cohort by phase of disease (14). Mittmann et al. (13) estimated the costs of home care for cases of colorectal cancer diagnosed between 2005 and 2009, and the costs of chemotherapy and radiation therapy for cases diagnosed between 2010 and 2015 (16). However, we are unaware of any prior publications from Ontario or any other jurisdiction stratifying health care costs after diagnosis of CRC by the presence or absence of a history of colorectal evaluative procedures during Period 1, or stratified by any other factor related to exposure to colorectal screening.

There are limitations to estimation of costs from administrative data, compared to prospective collection. There is the possibility of miscoding of diagnoses and procedures, which may occur more frequently in records completed by practitioners (e.g., OHIP physician billing claims database), compared to professional health records technologists (e.g., CIHI DAD and SDS databases), which in turn would distort the cost estimates. These estimates cannot be used to compute total government expenditures on the care of all CRC in Ontario by simple multiplication of the mean overall cost by the number of cases, due to the restrictive cohort definitions and exclusion criteria, which were required in order to meet the aims of this work. The estimates reflect the health care funding provided by the government of Ontario, and are not necessarily reflective of cost in other jurisdictions or in other currencies.

## Conclusions

Mean overall health care cost during the year following diagnosis of CRC is lower among cases who received colorectal evaluative procedures during Period 1 (10 years before the 6 month prediagnostic interval), generally with further evaluation during Period 2 (the 6 month prediagnostic interval), and among those with screening colonoscopy for hereditary CRC syndromes or affected first-degree relatives.
